# A penetrating ocular injury can affect the induction of anterior chamber–associated immune deviation

**Published:** 2008-02-11

**Authors:** Fang Lei, Junfeng Zhang, Jinsong Zhang, Hao He, Ying Du, Peizeng Yang

**Affiliations:** 1 Department of Ophthalmology, the First Affiliated Hospital, Department of Microbiology and Immunology, Zheng Zhou University, Zhengzhou, Henan Province, China; 2 State Key Laboratory of Ophthalmology, Zhongshan Ophthalmic Center, Uveitis Study Center, Sun Yat-sen University, Guangzhou, Guangdong Province, China

## Abstract

**Purpose:**

To determine the effect of penetrating ocular injury on the induction of anterior chamber-associated immune deviation (ACAID).

**Methods:**

An injection of 5 μl ovalbumin (OVA, 20 mg/ml) into the anterior chamber (AC) of female BALB/c mice was performed to induce ACAID. A penetrating ocular injury was induced via the limbus on OVA-inoculated eyes at 24 h, 48 h, 72 h, and 120 h following AC injection. The mice receiving an OVA inoculation without the ocular injury served as the AC-injection group. Delayed type hypersensitivity (DTH) was examined to evaluate the induction of ACAID. The levels of transforming growth factor (TGF)-β1, interleukin (IL)-10, and interferon (IFN)-γ produced by splenocytes were detected by enzyme-linked immunosorbent assays (ELISA). The frequency of CD4^+^CD25^+^Foxp3^+^T cells in the splenocytes was detected by flow cytometry.

**Results:**

A significantly decreased DTH response was observed in the AC-injection group as well as in mice that received a penetrating injury at 72 h and 120 h following AC-injection of OVA. The levels of TGF-β1 and IL-10 produced by splenocytes of mice in the AC-injection group and in the 72-h and 120-h group were significant higher than those in the 24-h and 48-h group. However, the levels of IFN-γ produced by splenocytes of the AC-injection group and the 72-h and 120-h group were significantly lower than those in the 24-h and 48-h group. An increased frequency of CD4^+^CD25^+^Foxp3^+^T cells was found in the AC-injection group and the 72-h and 120-h group.

**Conclusions:**

Penetrating ocular injury preformed shortly (24 h-48 h) after an AC injection of an antigen was able to abrogate ACAID and was associated with a decreased production of TGF-β1 and IL-10, an increased production of IFN-γ, and a decreased expression of CD4^+^CD25^+^Foxp3^+^T cells.

## Introduction

Systemic immune tolerance induced by injecting exogenous antigens (Ag) into the anterior chamber of the eye is called anterior chamber-associated immune deviation (ACAID) [1,2]. It is characterized by elevated serum levels of Ag-specific, noncomplement fixing antibodies, expanded clones of precursor cytotoxic T cells, and impaired Ag-specific delayed–type hypersensitivity (DTH) [3,4]. This phenomenon is important in protecting the eye from bystander damage of inflammation and in maintaining an immune privileged microenvironment within the eye [5,6].

The mechanisms involved in the development of ACAID have been studied for many years. Several factors, including the local immune microenvironment of the eye [7], the integrity of the eye-spleen axis [4,8,9], and the local anatomic integrity of the eye [10], have been shown to contribute to the induction of ACAID. Earlier studies have shown that enucleation of antigen-inoculated eyes three to four days after anterior chamber (AC) inoculation was able to prevent ACAID induction [11]. An AC injection of antigen using a 30-gauge needle and not 33-gauge needle could induce ACAID [12]. This suggested that a certain degree of injury leading to intraocular tumor necrosis factor (TNF) production was needed to induce ACAID. However, ACAID could not be induced in mice if corneal lesions were applied using an electrocautery [13]. The observations mentioned above suggest that the degree of ocular injury may have profound consequences on the expression of ocular immune tolerance. To further examine the mechanisms involved in maintaining ocular immune privilege, we developed a new experimental injury model in mice. Using this model, we show the involvement of CD4^+^CD25^+^Foxp3^+^T cells and three cytokines, transforming growth factor (TGF)-β1, interleukin (IL)-10, and interferon (IFN)-γ, in the development of ACAID.

## Methods

### Animals

Forty-eight specific, pathogen-free, female BALB/c mice (six to eight weeks old) were used in the experiments. Six mice were used in each group as listed below. Mice were purchased from the Guangdong Medical Laboratory Animal Center. All mice were treated according to the ARVO Statement for the Use of Animals in Ophthalmic and Vision Research.

### Induction of ACAID and preparation of penetrating ocular injury

**Figure 1:**
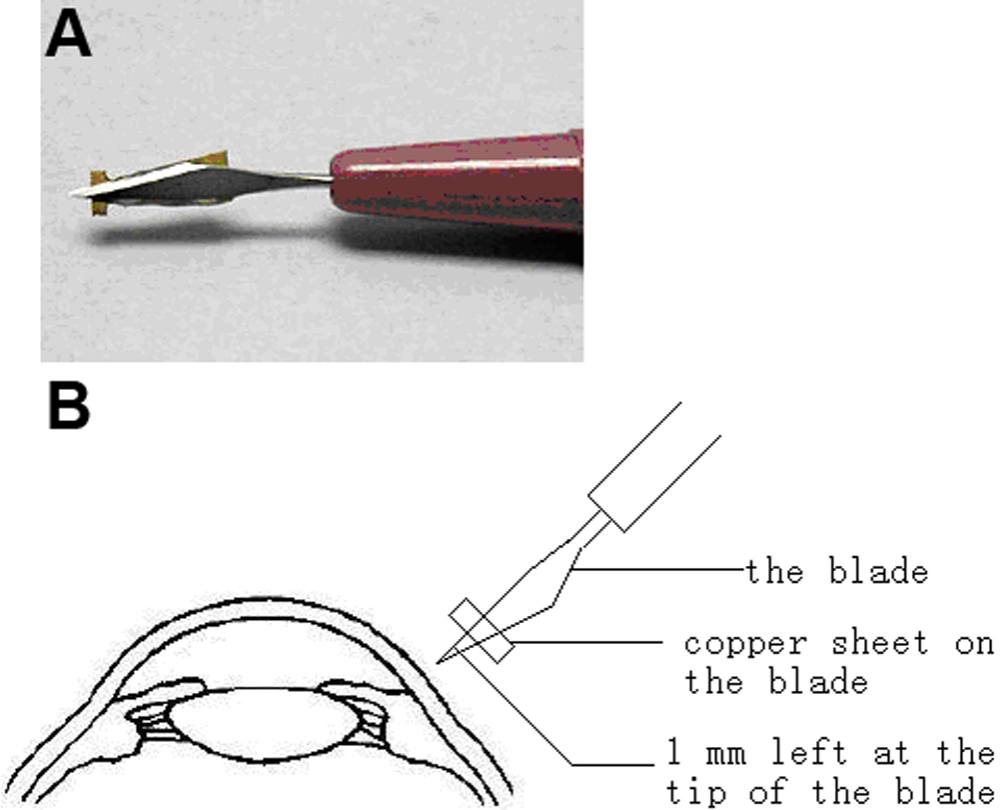
The method for preparation of a 1 mm-depth penetrating ocular injury at six o’clock of the murine limbus. A modified Standard Angle Blade is shown in the picture (**A**). A copper sheet was stuck 1 mm posterior to the blade tip. The diagram (**B**) showed that a 1 mm-depth penetrating ocular injury was made perpendicularly to the limbus using the modified standard Angle Blade.

ACAID was induced as described previously [14-16]. Mice were anesthetized with inhalation anesthesia consisting of oxygen and 1.7% isoflurane. Five microliters of ovalbumin (OVA; 20mg/ml; Sigma-Aldrich, Steinheim, Germany) dissolved in phosphate buffered saline (PBS) were injected into the AC of mice using a glass micropipette (about 80 μm in diameter) with a sterile infant feeding tube mounted onto a 0.1 ml Hamilton syringe (Hamilton, Reno, NV). Seven days later, the mice were immunized by subcutaneous (s.c.) injection of 250 μg OVA (2.5mg/ml, dissolved in PBS) emulsified 1:1 in complete Freund’s adjuvant (CFA; Sigma-Aldrich). This group of mice acted as the AC-injection group. Mice receiving an s.c. injection of OVA in CFA alone acted as positive controls. Untreated mice acted as normal controls. Mice receiving an injection of 5 μl sterile PBS into the anterior chamber and an s.c. injection of OVA in CFA seven days after AC injection acted as PBS controls. A penetrating ocular injury on OVA-inoculated eyes was performed 24 h (24-h group), 48 h (48-h group), 72 h (72-h group), and 120 h (120-h group) after AC injection. A 1 mm-depth penetrating ocular injury was made using a modified Standard Angle Blade (Alcan Laboratory, Fort Worth, Texas) as described below. A copper sheet was attached 1 mm posterior to the blade tip using metal gel to prevent a deeper injury (Figure 1A). Under a microscope, a perpendicular injury was carefully performed at the six o’clock position of the limbus using this modified blade (Figure 1B). Iris incarceration was observed in the eye immediately after this procedure had been performed. All mice were challenged with an s.c. injection of OVA in CFA seven days after the AC injection of OVA.

### Delayed type hypersensitivity assay

Seven days after s.c. immunization, the mice were challenged with an intradermal injection of 20 μl OVA (20 mg/ml) into the left ear pinna. The right ear pinna received an intradermal injection of 20 μl sterile PBS. Both ear pinnae were measured with a Mitutoyo engineer’s micrometer (MTI Corp, Paramus, NJ) before (0 h) and 24 h after the intradermal injection of OVA. The following formula was used to evaluate the DTH response: specific ear pinnae swelling= (24 h measurement-0 h measurement) of left ear- (24 h measurement- 0 h measurement) of right ear [17-19].

### Cytokine assay by enzyme-linked immunosorbent assay

Mice were sacrificed seven days after s.c. injections. Spleens were removed and grinded to prepare a single-cell suspension by density gradient centrifugation. The cells were incubated in a complete RPMI 1640 medium (Gibco, Grand Island, NY) with OVA (100 μg/ml for TGF-β1 measurement and 200 μg/ml for IL-10 and IFN-γ measurement) for 48 h in 24-well culture plates (2×10^6^ for TGF-β1 measurement and 4×10^6^ cells/well for IL-10 and IFN-γ measurement). The supernatants were collected for the measurement of TGF-β1, IL-10, and IFN-γ by enzyme-linked immunosorbent assays (ELISA) kits (R&D system, Minneapolis, MN).

### Detection of the frequency of CD4^+^ CD25^+^ Foxp3^+^ T cells in splenocytes by flow cytometry

Splenocytes were separated from the spleens of mice obtained from each group, and the frequency of CD4^+^ CD25^+^ Foxp3^+^ T cells was analyzed by BD FACSAria (BD Biosciences, San Jose, CA) using the BA FACDiVa software (BD Biosciences) in accordance with the manufacturer’s instructions. Briefly, 1×10^6^ splenocytes were first incubated with FITC-anti mouse CD3 monoclonal antibody (mAb; BD PharMingen, San Diego, CA), phycoerythrin (PE)-anti mouse CD4 mAb (eBioscience, San Diego, CA), and PE-cy7 anti mouse CD25 mAb (eBioscience) at 4 °C for 30 min. Cells were washed, fixed, and permeabilized using fixation/permeabilization buffer (eBioscience) at 4 °C for 1 h. Staining was performed with APC-anti mouse Foxp3 mAb (eBioscience) at 4 °C for 30 min, and the cells were washed, resuspended, and finally subjected to flow cytometry (FCM) for analysis. Cells stained with PE-cy7-conjugated rat immunoglobulin G1 (IgG1; eBioscience) or APC-conjugated rat IgG2a (eBioscience) served as isotype controls.

### Statistics

The results were expressed as mean ± standard error of the mean (SEM). Data were analyzed by analysis of variance (ANOVA) using SPSS 10.0. A value of p<0.05 was considered a significant difference.

## Results

### Delayed type hypersensitivity responses at different groups

**Figure 2:**
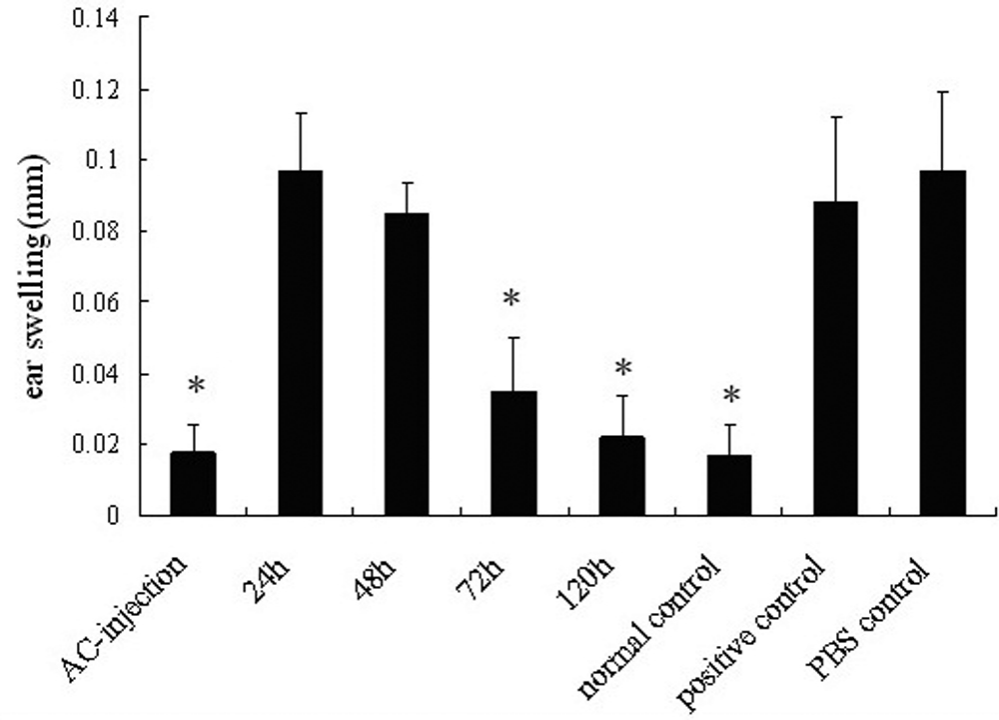
Delayed type hypersensitivity measurements were performed after an anterior chamber injection of ovalbumin. Mice that were immunized via an s.c. injection of OVA in CFA seven days after AC injection of OVA acted as the AC-injection group. Mice received a penetrating ocular injury 24 h (24-h group), 48 h (48-h group), 72 h (72-h group), or 120 h (120-h group) after an AC injection of OVA on the OVA-inoculated eyes. This was followed by an s.c. injection of OVA in CFA seven days after the AC injection of OVA. Mice without any treatment acted as normal controls. Mice immunized only with OVA in CFA acted as the positive controls. Mice receiving an AC injection of sterile PBS and an s.c. injection of OVA in CFA seven days after the AC injection acted as PBS controls. All mice received an ear challenge seven days after s.c. immunization. Both ear pinnae were measured before (0 h) and 24 h after the ear challenge. Mean ± SEM ear-swelling responses are presented (n=6 per group). The asterisk indicates that p<0.05 when compared with the positive control.

As the absence of a DTH response is a typical characteristic of ACAID [20], it was used as an index for the induction of ACAID in this study. The results showed that the DTH response was significantly suppressed in the AC-injection group and normal controls compared to the positive controls (both p<0.001). However, a marked DTH response comparable with that in positive controls was observed in animals that had received a penetrating ocular injury at 24 h and 48 h following AC injection of OVA but not in the 72 h or 120 h group. Similarly, a strong DTH response was also noted in the PBS control group (Figure 2).

### Expression of IFN-γ, TGF-β1, and IL-10 at different groups

**Figure 3:**
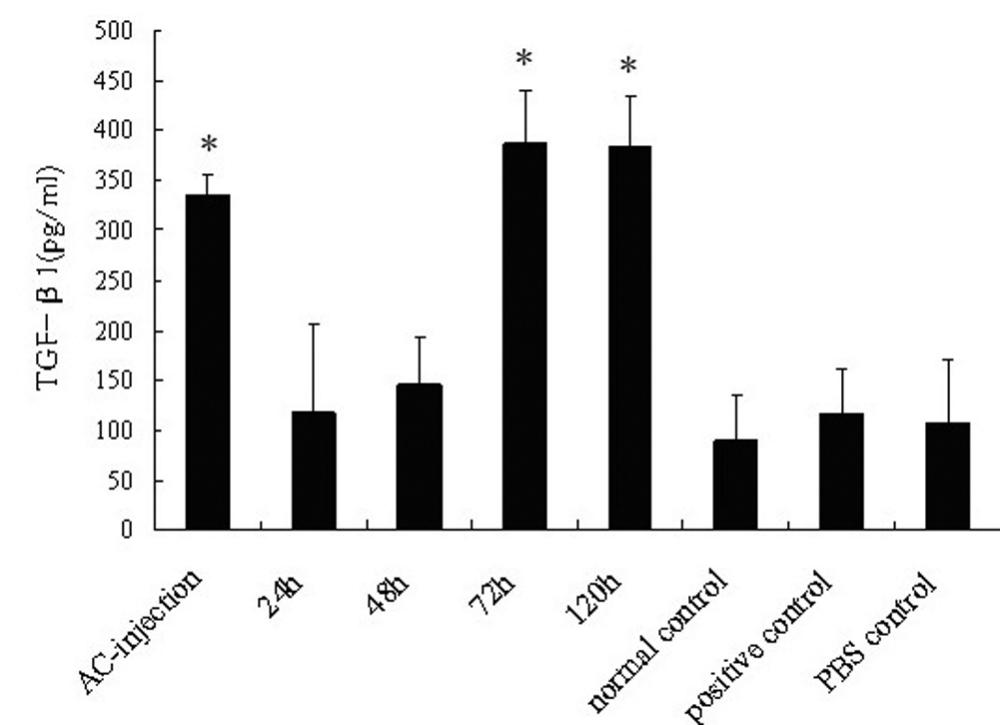
Detection of TGF-β1 produced by splenocyte. Mice were sacrificed seven days after s.c. immunization. Spleens were removed and grinded to prepare a single-cell suspension. These cells were incubated in a complete RPMI 1640 medium with OVA (100 μg/ml) for 48 h in 24-well culture plates (2×10^6^ cells/well). Supernatants were collected for determining the quantity of TGF-β1 by ELISA. Data are the mean ± SEM (n=6 per group). The asterisk indicates that p<0.05 as compared with the positive control.

**Figure 4:**
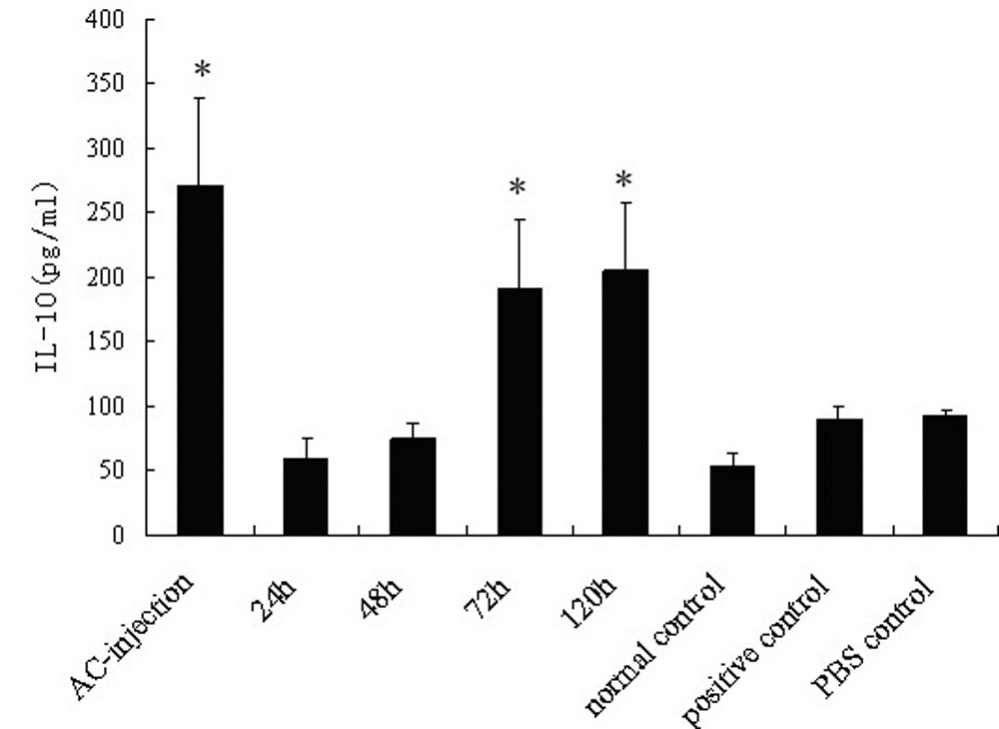
Detection of IL-10 produced by splenocytes. Spleens from each group of mice were harvested seven days after s.c. immunization and were used for preparing a single-cell suspension. The cells were incubated in a complete RPMI 1640 medium with OVA (200 μg/ml) for 48 h in 24-well culture plates (4×10^6^ cells/well). IL-10 in supernatants was quantified by ELISA. Data are the mean ± SEM (n=6 per group). The asterisk means that p<0.05 as compared with the positive control.

**Figure 5:**
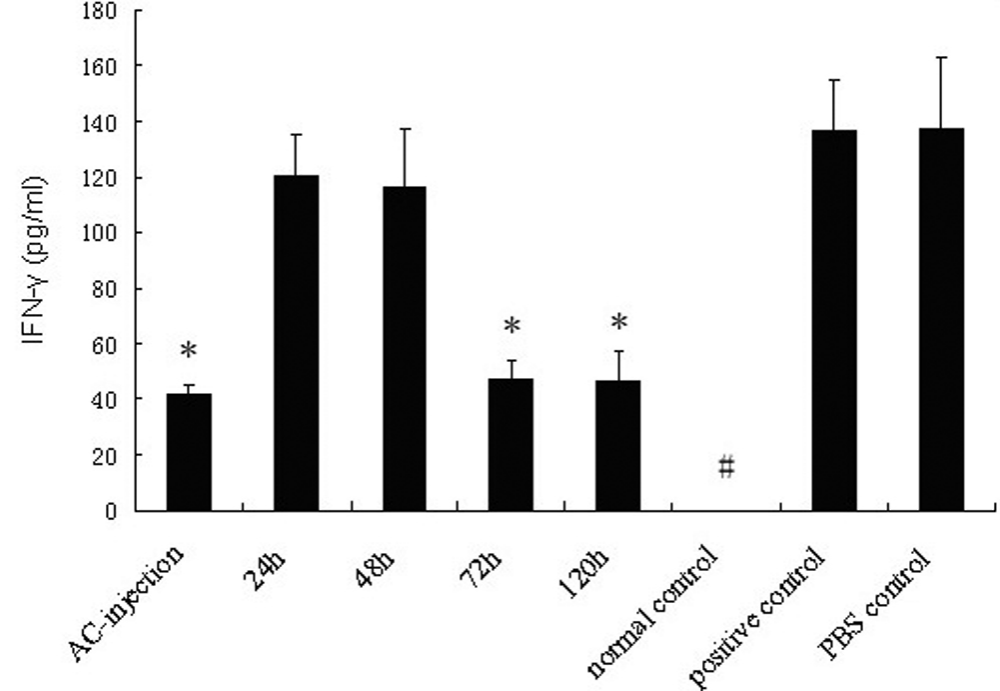
Detection of IFN-γ produced by splenocytes. Splenocytes were obtained from each group of mice seven days after s.c. immunization. The cells were incubated in a complete RPMI 1640 medium with OVA (200 μg/ml) for 48 h in 24-well culture plates (4×10^6^ cells/well). The levels of IFN-γ in supernatants were determined by ELISA. Data are the mean ± SEM (n=6 per group). The asterisk indicates that p<0.05 when compared with the positive control. The hash mark means that the production of IFN-γ was lower than the detection limit of the assay.

As cytokines, such as TGF-β1, IL-10, and IFN-γ, were involved in the induction of ACAID [21-25], our study examined whether there was an alteration in the production of these cytokines in the penetrating ocular injury groups. As shown in Figure 3, Figure 4, and Figure 5, the production of TGF-β1 and IL-10 was low and IFN-γ was undetectable in normal mice. An increased production of TGF-β1 and IL-10 and a low production of IFN-γ was observed in the AC-injection group compared to the positive controls (p<0.001, p=0.032, and p=0.005, respectively). The production of these cytokines was not different between the positive controls and PBS controls. A higher production of TGF-β1 and IL-10 and a lower production of IFN-γ were found in the 72-h and 120-h group. However, a decreased production of TGF-β1 (both p<0.001) and IL-10 (p=0.009, p=0.013, respectively) and an increased production of IFN-γ (p=0.001, p=0.008, respectively) were detected in the 24-h and 48-h group compared to the AC-injection group.

### Effect of ocular injury on the frequency of CD4^+^ CD25^+^ Foxp3^+^ T cells in splenocytes of ACAID mice

**Figure 6:**
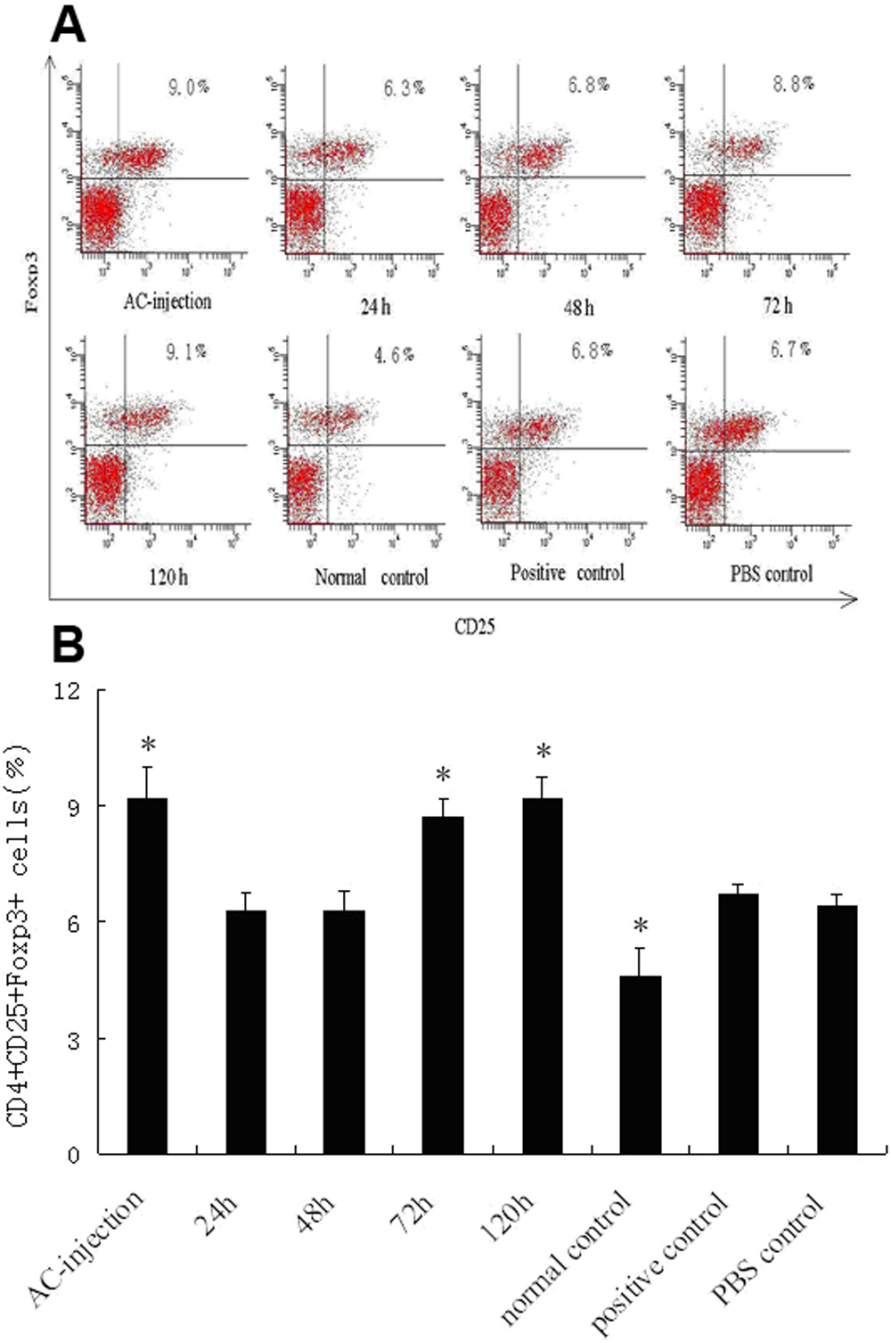
Flow cytometry analysis of CD4^+^CD25^+^Foxp3^+^T cells. Flow cytometry analysis of CD4+CD25+Foxp3+T cells per 1×106 splenocytes in mice from different groups. The mice in the groups listed as AC-injection 24h, 48h, 72h, 120h, normal controls, and PBS controls were as same as described in Figure 2. Spleens from different groups were removed 7 days after s.c. immunization and used for preparing a single-cell suspension. The cells were stained with anti-CD3, anti-CD4, anti-CD25, and anti-Foxp3 mAb. Data in **A** indicate the percentage of CD25+Foxp3+ cells which derive from gating on CD3+ CD4+ cells. The results shown in the histogram (**B**) are the mean SEM (n=6 per group). The asterisk indicates that p<0.05 when compared with the positive control.

An earlier study has shown that the CD4^+^ CD25^+^ Foxp3^+^ subpopulation of T cells play an important role in immune tolerance [26]. In this study, we examined the frequency of CD4^+^ CD25^+^ Foxp3^+^ T cells in splenocytes in ACAID mice with or without an ocular injury and compared the data with appropriate controls. Our results showed that a significantly higher frequency of CD4^+^CD25^+^Foxp3^+^T cells was observed in the AC-injection group as compared with the normal controls, PBS controls, and positive controls (all p<0.001). A result similar to that in the AC-injection group was also noted in animals that had received an ocular injury 72 h or 120 h following AC injection of OVA. However, the frequency of these cells did not increase in the 24-h or 48-h “injury” groups (both p<0.001 versus AC-injection group; Figure 6).

## Discussion

In this study, we injected OVA into the AC of mice and used the DTH response to evaluate the development of ACAID. The results showed that an AC injection of OVA could induce a decreased DTH response in association with an increased production of TGF-β1 and IL-10, an increased frequency of CD4^+^CD25^+^Foxp3^+^T cells, and a decreased production of IFN-γ. A penetrating ocular injury 24 h and 48 h following AC injection led to a marked DTH response, a downregulation of TGF-β1, IL-10, and CD4^+^CD25^+^Foxp3^+^T cells, and an upregulation of IFN-γ. These results suggest that a penetrating ocular injury at an early stage following an AC injection of OVA could prevent the induction of ACAID and that TGF-β1, IL-10, IFN-γ, and CD4^+^CD25^+^Foxp3^+^T cells may be involved in this process.

The ACAID model has received a great deal of attention in recent years mainly due to its physiologic and pathologic implications. Under normal conditions, antigens administered into the anterior chamber of the eye did not elicit a DTH response thereby protecting the eye from bystander damage of inflammation. However, ACAID can temporarily be circumvented by specific autoantigens (in combination with immunological adjuvants) or by following a systemic lipopolysaccharide injection that leads to severe intraocular inflammation [27]. The disruption of the integrity of the eye may also inhibit this immune tolerance and induce an intraocular inflammation. Sympathetic ophthalmia as seen in human patients is presumably mediated by an immune response to certain antigens (i.e., abortion of ACAID) due to the disruption of ocular integrity following penetrating ocular injury or intraocular surgery [28]. In this study, we examined whether and when a penetrating ocular injury influenced the induction of ACAID. As the absence of a DTH response is a crucial characteristic in ACAID, we evaluated the induction of ACAID by measuring the DTH response in mice following various treatments. The results showed that a DTH response was readily detected in positive controls and PBS controls but not in the AC-injection group or in the normal, untreated controls. A significant DTH response was also observed in the 24 h and 48 h “injury” group but not in the 72-h and 120-h group. These results indicate that the breakdown of the eye’s integrity at an early stage following an AC injection could induce a DTH response and was able to prevent the induction of ACAID. Our finding is consistent with experiments where the enucleation of an Ag-inoculated eye within 72 h of the AC injection prevented ACAID induction [29].

Earlier studies have shown that induction of ACAID is the result of a T helper cell 2 (Th2)-like response [30,31]. IFN-γ has been shown to inhibit the proliferation of Th2 cells, whereas IL-10 profoundly inhibits the production of Th1 cytokines [32]. It has been reported that an increased expression of TGF-β and IL-10 and a decreased expression of IFN-γ was associated with ACAID [31,33,34]. In this study, we examined whether a penetrating ocular injury influenced the production of these cytokines and if a relationship existed between the development of ACAID and the production of these cytokines. Our results showed that TGF-β1 and IL-10 were significantly upregulated while IFN-γ was markedly downregulated in the AC-injection group. These results are consistent with previously reported findings [31,33,34]. It is interesting to observe that a penetrating ocular injury 24 h or 48 h after an AC injection of OVA could lead to a decreased production of TGF-β1 and IL-10 and an increased production of IFN-γ, whereas a penetrating ocular injury at 72 h or later did not influence the profile of these cytokines. These results showed that there was a negative correlation between DTH response and the expression of TGF-β1 and IL-10 and that a positive association exists between DTH response and IFN-γ expression. These results suggest that the cytokines that are downregulated by a penetrating ocular injury during the early phase after an AC injection of OVA are probably involved in the development of ACAID. It is worthy to point out that an interesting result has been observed by Obta et al [35]. They found that IL-6 was able to prevent the development of ACAID when injected concurrently with an antigen into the anterior chamber. It has been shown that IL-6 is produced following viral/bacterial infection and corneal transplantation [36,37]. These results raise questions whether IL-6 is locally induced following a penetrating ocular injury and whether an increased level of IL-6 is involved in the abortion of ACAID. Further studies are needed to clarify these issues.

It has been reported that CD4^+^CD25^+^ regulatory T cells are important for the induction and maintenance of peripheral immune tolerance [38]. Foxp3 has been demonstrated as a specific marker for CD4^+^CD25^+^ regulatory T cells [39,40]. The frequency of CD4^+^CD25^+^Foxp3^+^T cells in splenocytes was significantly increased in ACAID [16]. In this study, we examined the frequency of these regulatory T cells in mice with or without penetrating ocular injury during the development of ACAID. It was found that the frequency of CD4^+^CD25^+^Foxp3^+^T cells in the AC-injection group was significantly increased, which was similar to the results reported by Zhu et al. [16]. However, the frequency of CD4^+^CD25^+^Foxp3^+^T cells was not increased in mice with penetrating ocular injury 24 h or 48 h following an AC injection of antigen. This result suggests that a penetrating ocular injury at an early stage after an AC injection of OVA does not alter the expression of CD4^+^CD25^+^Foxp3^+^T cells and thereby does not result in the induction of ACAID.

In conclusion, our study revealed that a penetrating ocular injury 24 h or 48 h after an AC injection of OVA could prevent the development of ACAID. It is likely that downregulation of TGF-β1, IL-10, and CD4^+^CD25^+^Foxp3^+^T cells and an upregulation of IFN-γ contribute to this aborted ACAID. However, our study did not answer the question as to how these alterations occurred following a penetrating ocular injury. We do not know whether the migration of tolerogenic dendritic cells (DC) from the eye to the spleen is altered or whether ocular DC are not tolerogenic due to the altered microenvironment within the anterior chamber following a penetrating ocular injury. It is also unclear whether other ways of injury could influence ACAID induction. More studies are needed to address these issues.
